# Barriers and Facilitators Associated with the Adoption of and Adherence to a Mediterranean Style Diet in Adults: A Systematic Review of Published Observational and Qualitative Studies

**DOI:** 10.3390/nu14204314

**Published:** 2022-10-15

**Authors:** Fotini Tsofliou, Dimitrios Vlachos, Christina Hughes, Katherine M. Appleton

**Affiliations:** 1 Department of Rehabilitation and Sport Sciences, Faculty of Health & Social Sciences, Bournemouth University, Bournemouth BH8 8GP, UK; 2 Centre for Midwifery, Maternal & Perinatal Health, Faculty of Health & Social Sciences, Bournemouth University, Bournemouth BH8 8GP, UK; 3 Department of Psychology, Faculty of Science and Technology, Talbot Campus, Bournemouth University, Fern Barrow, Bournemouth BH12 5BB, UK

**Keywords:** Mediterranean diet, MedDiet, barriers, facilitators, adoption, adherence, adults, systematic review

## Abstract

The Mediterranean diet (MedDiet) has been linked with physical and mental health benefits. Previous research, however, suggests that adoption and adherence to a Mediterranean diet might be difficult for people who live outside of the Mediterranean region. The aim of this systematic review was to investigate the factors that influence adoption and adherence to a Mediterranean style diet in adults aged 18 years old and over, as identified in published observational and qualitative studies. Following registration of our protocol on PROSPERO (ID: CRD42018116515), observational and qualitative studies of adults’ perceptions and experiences relevant to following a Mediterranean style diet were identified using systematic searches of databases: MEDLINE, the Cochane Library, CINAHL, Web of Science and Scopus, over all years of records until February 2022. A narrative synthesis was then undertaken. Of 4559 retrieved articles, 18 studies fulfilled our inclusion criteria and were included. Factors influencing adoption and adherence to a MedDiet were identified and categorized as: financial, cognitive, socio-cultural, motivational, lifestyle, accessibility & availability, sensory & hedonic and demographic. Similar barriers and facilitators are often reported in relation to healthy eating or the consumption of specific healthy foods, with a few exceptions. These exceptions detailed concerns with specific components of the MedDiet; considerations due to culture and traditions, and concerns over a cooler climate. Suggestions for overcoming these barriers and facilitators specific to adoption and adherence to the Mediterranean diet are offered. These data will inform the development of future studies of robust methodology in eating behaviour change which offer pragmatic approaches for people to consume and maintain healthy diets.

## 1. Introduction

The Mediterranean diet (MedDiet) reflects the typical traditional dietary pattern of the Mediterranean region. It is characterized by the daily consumption of fruit and vegetables, a high consumption of unrefined whole grains and pulses, and a high consumption of monounsaturated fatty acids (MUFAs), primarily from olive oil. It also includes a moderate consumption of fish and alcohol, predominantly in the form of red wine, a low-to-moderate intake of dairy products (usually in the form of yogurt and cheese), and a low consumption of poultry and red meats [1].

Observational research demonstrates a protective role for the MedDiet for many global health concerns, including cardiovascular disease (CVD), recurrent cardiac events and CVD mortality [2], metabolic syndrome [3], abdominal adiposity [1], and excessive gestational weight gain [4]. Further evidence justifying the promotion of the MedDiet for health benefit comes from randomized trials [2]. For instance, the PREDIMED study showed that a MedDiet supplemented with nuts could exert a beneficial effect on CVD risk and several secondary outcomes [5]. Studies during pregnancy reveal that greater adherence to a MedDiet may protect against offspring cardiometabolic risk [6], and prospective studies among pregnant women, showed that low MedDiet adherence was associated with higher blood pressure and preeclampsia risk [7,8].

While health benefits are recognised however, adoption and adherence to the MedDiet outside of the Mediterranean region entail difficulties and rates of adherence are decreasing in Mediterranean and southern Europe [9]. Studies suggest various barriers to the consumption of a Mediterranean style diet, but a comprehensive account may be informative. This work aimed to identify all studies investigating any barriers or facilitators to adopting or adhering to a Mediterranean style diet, in order to obtain an evidence-based insight of reasons that influence MedDiet adoption and adherence. To the best of our knowledge, no systematic review has yet been undertaken with this aim.

## 2. Materials and Methods

This review was conducted following the Centre for Reviews and Dissemination (CRD)’s guidance for undertaking reviews in health care [10]. Barriers and facilitators to following a MedDiet were compiled according to defined outcome categories. Methods and inclusion/exclusion criteria were determined in advance and a protocol for the review was developed and sent to an advisory group of the International Prospective Register of Systematic Reviews (PROSPERO). The protocol was published in PROSPERO on 23 November, 2018 (registration no. CRD42018116515).

### 2.1. Systematic Search Strategy

Search terms were generated by team discussion and an initial review of the literature. Relevant words were combined using logical ANDs and ORs to create one search string that included terms related to “Mediterranean diet”, “barriers”, “facilitators” and “adults”. This search string was: (“Mediterranean diet”* OR med-diet OR MD OR “Mediterranean style diet”* OR “Mediterranean-style diet”*) AND ((Barrier* OR obstacle* OR difficult*) OR (enabler* OR facilitator* OR factor* OR reason* OR determinant* OR motivator* OR characteristic*)) AND (adult* OR mature* OR elder* OR aged).

Electronic databases: MEDLINE, the Cochrane library, CINAHL (Cumulative Index to Nursing and Allied Health Literature), Web of Science and Scopus were investigated from inception by searching in “title”, “abstract” and “keywords” fields until February, 2022. Searches were not limited by study design, but they were limited by language of publication, where only English articles were considered. Search results were exported into EndNote, duplicates were deleted, and the remainder imported into COVIDence (www.COVIDence.org (accessed on 1 January 2019)) [11]. All studies were initially independently screened on titles and abstracts against the eligibility criteria by two researchers (FT, DV) with no conflicts. Full texts for all potentially eligible papers were obtained. Citation tracking and reference lists of all retrieved articles were also reviewed by hand for any further eligible articles.

### 2.2. Inclusion and Exclusion Criteria

The review included only observational and qualitative studies. Any observational (e.g., cross-sectional, cohort) study was acceptable. Studies were included if they reported on factors that influence the adoption of or adherence to a Mediterranean style diet or a dietary intervention that includes similar food patterns, in adults aged 18 years and older. Only peer reviewed full journal papers, published in English, were included. Studies were excluded if they focused on children or adolescents; if they focussed on diets other than the MedDiet, such as a vegetarian diet or PALEO diet; if they included only single components of a MedDiet (where we considered “fruit and vegetables” as a single component), and if they were not published in English. The inclusion and exclusion criteria were piloted by three researchers (FT, DV, KA). Then, the assessment was performed for the full list of identified studies by two researchers (DV, FT). Disagreements were resolved between the two researchers or by consultation with a third author (CH or KA).

### 2.3. Data Extraction

Data from all studies were subsequently extracted by two researchers (FT, DV) using the COVIDence tool, an online tool designed to streamline the process of conducting a systematic review [11]. Data on each study included details of country of origin, participant demographics, sample size, data collection method and outcomes. Data on factors influencing adoption or adherence to the MedDiet included all reported barriers and facilitators. Factors were categorized as: availability/accessibility (relating to procurement); cognitive (relating to knowledge, thoughts and understanding); demographic (relating to gender, age, socio-economic status); financial (relating to cost and financial concerns); lifestyle (relating to other lifestyle characteristics, e.g., physical activity, smoking habits); motivational (relating to willingness); sensory & hedonic (relating to sensory aspects of foods, including liking and pleasure); and socio-cultural (relating to societal and cultural concerns). All barriers and facilitators were included in the review, regardless of the frequency/number of respondents who reported them. Other researchers (CH, KA) independently checked the extracted data for accuracy and completeness, and uncertainties were resolved by discussion. Uncertainties considered the categorisation of barriers and facilitators, and variations in reporting style between papers.

### 2.4. Risk of Bias

All included studies were also assessed for risk of bias. Risk of bias in observational studies was evaluated using the STROBE Statement for Observational Studies in Nutritional Epidemiology [12] and risk of bias in qualitative studies was evaluated using the Critical Appraisal Skills Programme (CASP) tool [13]. Two authors (FT, DV) independently evaluated the included studies, and any discrepancies were resolved by discussion. Final judgements were summed to provide a total score.

## 3. Results

**Figure 1:**
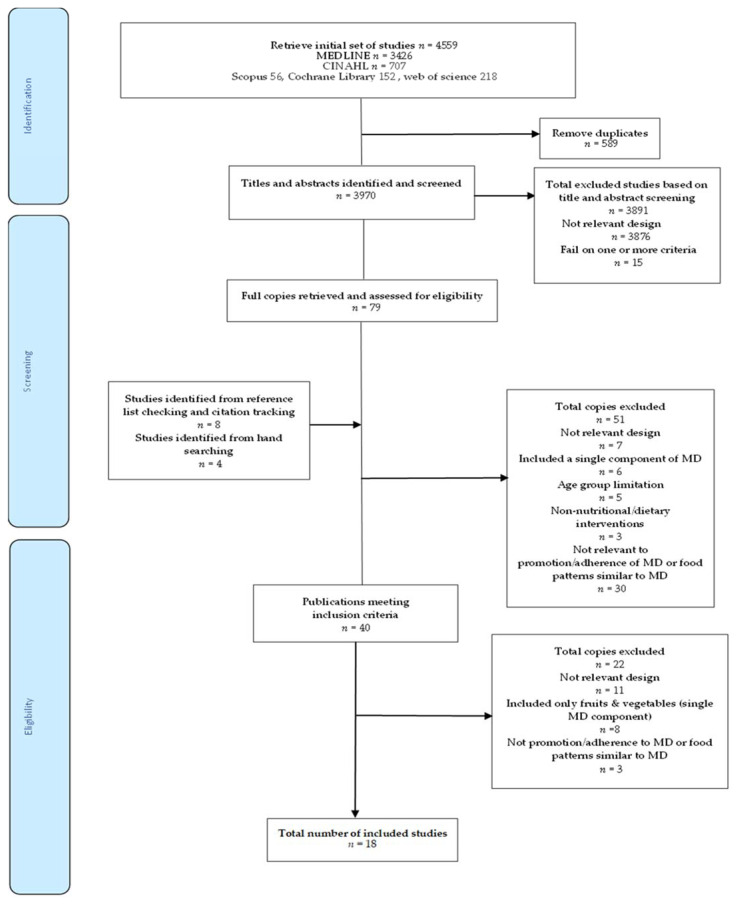
Preferred Reporting Items for Systematic Reviews and Meta-Analyses (PRISMA) flow diagram of the search strategy and study selection process.

Searches were most recently conducted on 22 February 2022 to result in the identification of 4559 citations, of which 79 were screened on full text. In total, 18 studies were included in the review. The detailed study selection process is shown in Figure 1.

### 3.1. Characteristics of Included Studies

Characteristics of the included studies and ratings of risk of bias are shown in Table 1 and Table 2. Of the 18 included studies, 12 were observational studies, all cross-sectional in design [14,15,16,17,18,19,20,21,22,23,24,25], and 6 were qualitative studies [26,27,28,29,30,31]. Four studies, all qualitative [26,27,28,30] were related to adoption of MedDiet and 14 studies, both qualitative [29,31] and observation studies [14,15,16,17,18,19,20,21,22,23,24,25] were related to adherence to MedDiet. Four studies were based in Mediterranean regions—one each from Italy [14] and Greece [17], and two from Spain [15,16], and all others were conducted in non-Mediterranean regions. Seven of these studies were conducted in the United Kingdom [22,23,25,26,28,29,30] four studies were conducted in the USA [17,18,20,21], two studies were conducted in Australia [24,31] and one was conducted in the Netherlands [19].

Sample sizes ranged from 11 participants [29] to 67 participants [30] for the qualitative studies and from 236 participants [17] to 36,032 participants [14] for the observational studies. Two studies included females only [16,28].

### 3.2. Findings of Included Studies

Barriers and facilitators in all eight categories are given in Table 3. Many barriers and facilitators were reported, and barriers and facilitators were identified within all eight categories.

In relation to availability and accessibility, barriers included difficulties in accessing suitable foods, due to limited availability, choice and possibly due to season, and facilitators included good access to foods in general, e.g., due to good access to preferred retail outlets and suitable food provision in catering outlets. Linked to accessibility, high food costs were also reported as barriers to MedDiet consumption, high relative food costs (high food insecurity) or the increased consumption of foods that are perishable and so may result in increased food waste. Conversely however, financial facilitators included consideration that the MedDiet is good value for money and the recognition that many foods that are consumed in large quantities within the diet are relatively cheap, while more expensive food items, such as meat, should be consumed less often. Low income was also given as a financial barrier, as was a lower level (non-managerial/non-professional) occupation. Gender, age and education were other demographic barriers and facilitators, such that following a MedDiet was more likely in females, older individuals, and those with a higher education. Related lifestyle characteristics included physical activity habits, smoking habits, obesity and the presence of medical concerns and conditions.

In the cognitive domain, barriers included a lack of knowledge of the MedDiet, a lack of knowledge of the details of the diet, e.g., which specific foods were included and how these could be incorporated into meals, and a lack of knowledge of the value of the diet for health, or confusions and concerns over the health implications of some of the food components. Facilitators included perceptions of improved diet quality, including the consideration of naturalness, a range of health benefits and positive outcomes, affecting physical health, body-weight and well-being, and some environmental benefits. Sensory and hedonic barriers and facilitators focused on the taste, smell and pleasure to be gained from recommended foods, the loss of pleasure as a result of giving up foods that should be consumed in lower quantities, and degree of familiarity with the recommended foods. Motivational facilitators included factors such as high self-efficacy, high self-regulation and willingness to change, while motivational barriers considered the reverse, plus some concerns over restriction. Socio-cultural barriers and facilitators included aspects of the individual’s family and living circumstances, type of upbringing, aspects of the individual’s lifestyle and situation, e.g., working night shifts, time available for cooking, cooking skills, and aspects of the wider culture, including the climate. In relation to the wider culture, cultural differences, such as traditional meal patterns and traditional dining patterns were reported as barriers, and changes to these patterns were reported as facilitators. Similarly, a cold climate was reported as a barrier, while a warm climate was a facilitator.

## 4. Discussion

The present research aimed to systematically identify and summarize all published observational and qualitative studies that investigated barriers and facilitators influencing adoption and adherence to a Mediterranean style diet. Our searches identified twelve observational studies, and six qualitative studies. A number of barriers and facilitators were found for each of the categories.

These barriers and facilitators are largely reported also in relation to healthy eating, or the consumption of specific healthy food items, such as fruits and vegetables. Poor availability and accessibility are a commonly reported barrier to healthy eating [31,32], but specific foods that form part of the MedDiet may incur specific concerns. There can be limited access to a range of MedDiet components in shops and supermarkets in non- Mediterranean regions, for example [2].

Financial concerns are also frequently reported as barriers to healthy eating [32,33,34,35,36], alongside perceptions that healthier diets or dietary patterns closer to dietary guidelines are more expensive or poorer value for money due to lower energy content or likely food waste [32,34,37]. A Mediterranean dietary pattern has even been explicitly demonstrated as more expensive than some Western diets [35,38]. Higher household income is also often positively associated with higher diet quality in adults [39], while lower household incomes have been consistently associated with poorer diet quality [34,40]. Some research also suggests higher costs to be associated with the consumption of specific key components of the MedDiet [38], but Goulet et al. [41] using a nutritional intervention promoting a Mediterranean food pattern in North America, found a negative association between adherence to MedDiet and an increase of daily food expenses.

Healthy eating is well recognised as socially patterned, based on gender, age, socio-economic status, income and education [42,43,44,45,46,47], and tends to co-exist with other healthy lifestyle behaviours, such as non-smoking and higher levels of physical activity [15,44,45,46]. Cognitive factors, such as a lack of knowledge or concerns and confusions over knowledge [37,48,49], motivational factors, such as a lack of willpower [22,33,36,37,45], and sensory or hedonic factors [22,33,34,36,43,45,48] are also frequently reported. Specific challenges to following the MedDiet in this respect, might be low liking or a low familiarity with some of the specific tastes and food items to be included.

Living situation or aspects of the living situation, such as an unsupportive partner, too little time or inadequate cooking skills are also relevant to healthy eating [24,32,33,34,36,37,45], but again specific concerns may arise over some of the specific food components of the MedDiet. The MedDiet, for example, is high in pulses and legumes, and lack of knowledge or confidence in how to best prepare pulses has been found to hinder the regular consumption of pulse-centric diets [50]. Concerns over cultural differences, such as changes to traditional meal patterns and traditional dining patterns and perceptions around unsuitable climate may similarly by specific to adoption and adherence to the MedDiet. Previous research also highlights the difficulty of the transferability of the MedDiet to non-Mediterranean countries [3].

Barriers and facilitators that are specific to the MedDiet, thus, detailed concerns with specific components of the MedDiet, considerations due to culture and traditions, and concerns over a cooler climate. Components of the MedDiet of concern included the high amount of olive oil to be consumed, which can be expensive and was considered to potentially lead to weight gain, the high consumption of pulses and legumes, which can be time-consuming and difficult to cook, or can be difficult to make tasty and pleasurable, the moderate consumption of fish, which for some people is unappealing and the low consumption of red meat which again for some can be unappealing. Suggestions to overcome knowledge-based concerns include education on the value of all food components for health and wellbeing [3,50,51], and on the value of all foods combined [3,51]. Benefit may be gained particularly from education on benefits for body weight or appearance [31,33,37]. Suggestions to overcome financial concerns may also lie in education, on the low cost of some MedDiet components, such as pulses and legumes [41,51], or on possible alternatives to some food components to create an adapted version of a Mediterranean style diet, e.g., through the use of locally available rapeseed oil and nuts [51,52,53]. Suggestions to overcome sensory and hedonic concerns may again benefit from increased choice in local markets, for fish, fruits and vegetables, plus the promotion of recipes and tastings [51]. Suggestions to overcome practical concerns include the increased availability of pre-prepared foods, the promotion of simple and easy recipes [36,50,51], and the promotion of cooking classes and skill development [34,36,50].

In relation to culture, tradition and climate, few changes can be made to these external factors, but suggestions can again be made to change perceptions towards them. Recipes for warm dishes, for example, have been suggested to encourage the perception that the MedDiet is suitable for cooler climates [30,51]. Use of substitutions for some food components will increase perceptions of flexibility and allow perceived differences to be reduced [52,53]. Focus on the similarities between the eating patterns in different countries may also be helpful. Some tailoring specific to cultures, traditions and individual tastes may also be beneficial.

Strengths of the review include the use of systematic searches and processes, such that all processes were undertaken by two authors independently. The studies found represent the perceptions and experiences of over twenty-five thousand men and women from nine different countries. As limitations, the review only considered studies written in English, and focused only on healthy adults, and as a result, the findings may not be transferable to non-healthy and underage populations, or to some locations. We also did not search for unpublished studies, and only four studies were found that had been conducted within the Mediterranean region. Further studies would have allowed distinction between barriers and facilitators based on geographical location, and may have provided some insights specifically in relation to location and culture.

## 5. Conclusions

This synthesis of observational and qualitative studies provides insight into the barriers and facilitators that can influence adoption and adherence to a Mediterranean Diet in healthy adults. A range of barriers and facilitators were found. Similar barriers and facilitators are often reported in relation to healthy eating or the consumption of specific healthy foods, with a few exceptions. These exceptions detailed concerns with specific components of the MedDiet, e.g., the weight implications of a high fat consumption through increased oil and nut use, the cost of olive oil, the preparation time and skills required for consuming pulses; considerations due to culture and traditions, and concerns over a cooler climate. Suggestions for overcoming these barriers and facilitators specific to adoption and adherence to the Mediterranean diet are offered. These data will inform the development of future studies of rigorous designs and robust eating behaviour methodology, which can offer pragmatic approaches for people to consume and maintain healthy diets.

## Figures and Tables

**Table 1:** Observational studies investigating barriers or facilitators to adopting or adhering to a Mediterranean style diet.

Authors	Location	Aims	Participants	Data Collection Method	Risk of Bias
Mediterranean Countries					
Cavaliere et al., 2019 [14]	Italy	To analyse whether socioeconomic status (SES) is ultimately related to the overall level of adherence to the Mediterranean Diet (MD) of the Italian population	*n* = 36,032 Males and females aged ≥18 years	Italian household food survey questionnaire and adherence to MD pyramid recommendations by a study-based index	21/22
Moreno-Gomez et al., 2012 [15]	Spain	To ascertain the prevalence of and association between main lifestyle factors such as diet, physical activity, alcohol consumption, smoking in students	*n* = 987 Males and females Age (mean (SD) 21.4 (3.3)	Modified Mediterranean diet score adapted to diet patterns and needs of study’s population group	21/22
Olmedo-Rquena et al., 2014 [16]	Spain	To investigate the factors associated with the level of adherence to a Mediterranean dietary pattern in childbearing women before pregnancy	*n* = 1175 Females aged ≥18 years	Structured survey with FFQ and Mediterranean diet adherence index specific to Spanish MD guidelines	22/22
Thodoridis et al., 2018 [17]	Greece	To associate MedDiet adherence and food insecurity among university students in Greece	*n* = 236 Males and females aged 19–30 years	Household Food Insecurity Access Scale for MD barriers and MD adherence assessed with MEDAS questionnaire	21/22
Non-Mediterranean Countries					
Couto et al., 2021 [18]	USA	To examined MedDiet adherence and perceived knowledge, benefits, and barriers to the MedDiet in a Portuguese immigrant community	*n* = 208 Males and females 45 ≥ 75 years	Self-reported survey questionnaire on barriers and MEDAS score	22/22
Dijkstra et al., 2015 [19]	NL	To identify barriers for meeting the fruit, vegetable and fish guidelines in older Dutch adults and to investigate socio-economic status (SES) differences in these barriers	*n* = 1057 Males and females 55–85 years mean age of 68.9 (SD 6.2) years	Self reported food frequency questionnaire for MD adherence and lifestyle questionnaire	21/22
Greiner et al., 2018 [20]	USA	To examine adherence to the Mediterranean diet in patients diagnosed with cardiovascular disease based on social cognitive theory constructs	*n* = 337 Cardiac patients	Validated Food beliefs questionnaire and study-based 7-item MedDiet score	22/22
Knight et al., 2019 [21]	USA	To examine MedDiet adherence and perceived knowledge, benefits, and barriers to the MedDiet in the U.S.	*n* = 1447 Males and females, aged >18 years	Online questionnaire survey and MedDiet adherence evaluated by validated 14-point Mediterranean Diet Adherence Screener (MEDAS)	22/22
Lara et al., 2014 [22]	UK, England	To evaluate the association between perceived barriers to healthy eating and adherence to MD	*n* = 206 Males and females aged ≥50 years	Online Questionnaire-survey of 14-point Mediterranean Diet Adherence Screener (MEDAS)	21/22
Papadaki et al., 2015 [23]	UK, England	To assess internet usage patterns and adherence to the MedDiet among employees in South-West England, UK and their differences by personal characteristics	*n* = 590 Males and females mean age 43.8 years	Self-reported food frequency questionnaire of 11 main components of the Mediterranean diet	22/22
Scannell et al., 2020 [24]	Australia	To investigate the perceived beliefs, barriers, and enablers toward adherence to a MedDiet in Australian adults	*n* = 606 Females and males aged ≥18 years	Self-reported online questionnaire on barriers and adherence to a MedDiet via validated 14-item Mediterranean Diet Adherence Screener (MEDAS)	22/22
Tong et al., 2018 [25]	UK, England	To examine the dietary cost associated with adhering to the MedDiet in the United Kingdom and to assess the extent to which this association is influenced by socio-economic factors	*n* = 12417 Males and females 30–65 years	Self-reported food frequency questionnaire and study based Mediterranean diet score (MDS)	22/22

**Table 2:** Qualitative studies investigating barriers or facilitators to adopting or adhering to a Mediterranean style diet.

Authors	Location	Aims	Participants	Data Collection Method	Risk of Bias
Non-Mediterranean Countries					
Haigh et al., 2019 [26]	UK, England	To identify factors that affect Mediterranean diet adoption (and adherence/maintenance) in a northern European NAFLD population.	*n* = 19 Females, Age (mean(SD) 58.5 (10.6)	Semi structured interviews and MedDiet adherence via validated 14-point Mediterranean Diet Adherence Screener (MEDAS)	10/10
Hardin -Faning, 2013 [27]	USA	To identify factors that affect adoption and future adherence to a MedDiet in a rural Appalachian food desert	*n* = 43 Males and females aged ≥21 years	Self-reported open-ended questions	9/10
Kretowicz et al., 2018 [28]	UK, England	To investigate the perceived barriers to following a MedDiet in women of childbearing age	*n* = 20 Females aged 18–49 years	Focus groups and MEDAS	9/10
Middleton et al., 2015 [29]	UK, England	To examine the participants’ experiences regarding perceived barriers and facilitators which impact on consuming the MedDiet	*n* = 11 Males and females aged 50–65 years	Semi-structured focus groups	9/10
Moore et al., 2017 [30]	UK, Northern Ireland	To investigate attitudes towards adoption of a MedDiet in individuals at high CVD risk in a Northern European population	*n* = 67 Males and females, Age (mean (SD) 64.0 (10.0)	Semi-structured focus groups and adapted eight-item Food Frequency Questionnaire (FFQ) based on MEDAS	10/10
Zacharia et al., 2020 [31]	Australia	Τo assess perceived barriers and enablers in order to support older Australians to adhere to an AusMed diet pattern	*n* = 6 Males and females aged over 55 years	Individual Semi-Structured Interviews	8/10

**Table 3:** Perceived Barriers and Facilitators to adoption or adherence to Mediterranean diet.

Themes	Barriers	Facilitators
Availability/Accessibility	Difficult to purchase food items (e.g., little support to find and locate items in supermarket) [29,30] Seasonal availability of foods [24,28] Limited access, selection and choices [19,24,27]	Increased access to fresh foods [24] Availability of diet in catering outlets [24] Good access to budget supermarkets and good transport [26]
Cognitive	Absence of nutrition education (e.g., limited knowledge of the health benefits of MedDiet foods) [18,19,21,27,28,30] Lack of knowledge of how to incorporate the food components of the MedDiet into meals [24,27] Complex & contradictory dietary information (e.g., conflicting reports from media) [28,30] Complexity and size of meal plans [31] Concerns over specific foods (e.g., fats & oils) [28] Concerns over food safety (e.g., pesticide residues) [19] Low food literacy [24] Low health appeal and perceived healthfulness [18,21,24] Negative perceived outcomes, e.g., increased body weight [20,30] Lack of understanding of the importance of nutrition [26] Nutritional attitudes and beliefs [26,28]	Good nutrition knowledge and skills [26] Improved diet quality [24,29] Physical benefits, e.g., disease prevention [24,26,28,29] Appearance/body weight related benefits, inc. weight loss [18,21,26,28] Psychological benefits, e.g., improved well-being, mood [18,28] Natural content [18] Environmental benefits [24] Recipes to enable food incorporation [24] Understanding of links between diet and disease [26] Nutritional attitudes and beliefs [26] Trusted advice [26] Positive perceived outcomes [20]
Demographic	Lower education level [14,16,19,21] Lower SES [16] Gender: Being male [14] Gender: Males were less likely to understand the health implications of the MedDiet and less amenable to making chages [27] Age: Younger [14,16] Age: Younger people were concerned about the cost [27]	Higher education attainment [16,18,23,25] Lower education attainment [18] Gender: Being female [14,15,22] Age: Being older [16]
Financial	Increased food costs [19,24,27,28,29,30,31] Greater food expenses on high consumption items, e.g., legumes, Lower food expenses on low consumption items, e.g., meat [25] Wasteful/Foods easily spoil (e.g., fruits and vegetables) [19,24] Poor storage facilities [19] Lower incomes [14,19,27] High food insecurity [17]	Higher household income [25] Occupation (Professional/managerial) [25] Relative expense of plant-vs animal based foods [28] Good value for money [18,21,24,26]
Lifestyle	Sedentary lifestyle [16] Habit of smoking [16,17] Greater number of previous pregnancies [16] Medical concerns/poor health [19,26] Higher BMI [21]	Active lifestyle [16] Lower BMI [22] Higher number of meals consumed daily [15]
Motivational	Lack of willpower/motivation to cook healthy foods [24,26] Resistance to dietary change [27,30] The term “diet” elicited ideas of restriction [28] Low abilities to adhere, due to the restrictive nature [24] Low willingness for self-care [26] Perceived helplessness [26] Impacts of poor adherence [26] High responsiveness to external food cues [25]	High self-efficacy/self-determination [20,26] Good self-regulation to consume healthy foods and avoid unhealthy foods [20,26] Higher stage of dietary change [18,21] Desire to increase healthy choices [24] High motivation [26] Balanced relationship with food [26]
Sensory and Hedonic	Low sensory appeal [18,21] Not liking the taste/smell [19,24,26] Trouble with chewing fruits (especially in older adults) [19] Components of the diet were unappealing (e.g., lentils) [28,31] Finding it hard to give-up liked foods [28] Poor appetite [19]	Enjoyable and pleasurable eating experience (i.e., sense of pleasure and fulfilment to meal times) [26,29] High taste/sensory appeal [18,21,24,26,31] Satisfaction (fillingness) with the foods [31] Varied [26,28] Familiarity with foods [18,21]
Socio-cultural	Negative influence of family members/dining partners [19,26,27,28] Upbringing and family [28] Usual habits [19,26,28] Acceptability of a MedDiet: Difficulties in adapting a new eating pattern and changing personal established eating habits [24,29,30] Stress, stressors, work and time pressures (e.g., irregular working hours) [26,29] Working night shifts [17] Impractical [18,19,21,28] Culture and cultural differences (e.g., British culture has a tradition of eating a lot of red meat) [24,26,29,30] Perceived difficulty of living in a colder climate (e.g., some food patterns of MedDiet are being eaten cold and people prefer warmer foods) [28,29,30] Not convenient to prepare and cook fresh food [26,27,28] Lack of cooking skills/equipment [24,26,27,28,30] Time to plan, purchase and prepare foods [24,27,28,29,30] References to mothers as responsible for their offspring [28] Climate—cold weather [24] Obesogenic environment [26]	Being married or cohabiting [14,23] Family/friend support [24,26] Shared responsibility of food preparation [23] Upbringing and family [28] Broadens the food repertoire [29] Redefines cultural eating habits, e.g., eating together [24,29] Increased time availability [24] Increased cooking skills/equipment [24] Climate—warm weather [24] Coverage in the media [26] Higher number of meals/day [15]

## Data Availability

Not applicable.
